# Positioning laboratory automation for today's dynamic climate

**DOI:** 10.1155/S1463924694000283

**Published:** 1994

**Authors:** D. G. Vogt

**Affiliations:** Eli Lilly and Company Lilly Corporate Center Indianapolis Indiana 46285 USA

## Abstract

Laboratory automation has existed and matured at Eli Lilly and Company for well over a decade. The author's section serves as a developer of laboratory automation systems for customers within Lilly and embodies ‘robotic friendly’ laboratories with highly technical and experienced personnel. With several systems showing signs of age, second generation ‘smart systems’ have been developed and delivered during the last three years. These systems were built with an ideology different from previous systems. Upon their delivery, the ‘smart systems’ met the customer's functional requirements but the overall acceptance of this ideology is still being debated due to the perception of failure. Much of this perception can be attributed to the delivery of a system heavily dependent on system maintenance, something totally unexpected by the customer. This paper discusses the ideology of a‘smart systems’ and the results following implementation. The events that led to the review and subsequent departure of the ‘smart systems’ ideology are also described.


Journal of Automatic Chemistry, Vol. 16, No. 6 (November–December 1994), pp. 231–233

### Positioning laboratory automation for today's dynamic climate

**D. G. Vogt**
*Eli Lilly and Company, Lilly Corporate Center, Indianapolis, Indiana 46285, USA*

*Laboratory automation has existed and matured at Eli Lilly and Company for well over a decade. The author's section serves as a developer of laboratory automation systems for customers within Lilly and embodies 'robotic friendly' laboratories with highly technical and experienced personnel.*

*With several systems showing signs of age, second generation 'smart systems' have been developed and delivered during the last three years. These systems were built with an ideology different from previous systems. Upon their delivery, the 'smart systems' met the customer's functional requirements but the overall acceptance of this ideology is still being debated due to the perception of failure. Much of this perception can be attributed to the delivery of a system heavily dependent on system maintenance, something totally unexpected by the customer. This paper discusses the ideology of 'smart systems' and the results following implementation. The events that led to the review and subsequent departure of the 'smart systems' ideology are also described.*

### History

Laboratory automation at Eli Lilly and Company has grown tremendously over the past decade. During this period, the author's group has developed over 10 robotic sample preparation systems for internal customers. With the development of these systems has come the development of robot friendly laboratories. Hamilton [1] has previously described the flexible robot friendly laboratories now commonplace within the author's section. These laboratories include 'raised floors over flexible services, service drops from ceiling bulkheads, quick-connect services, movable walls and movable benches [2].'

Brunner [3] and Hamilton [2] both have discussed the importance of having the appropriate personnel involved in the automation effort. Over the last several years, the author's group has acquired a proper mix of personnel with mechanical, electrical, computer programming, and chemistry backgrounds. This set of skills has greatly facilitated the automation effort by adding tremendous flexibility in each phase of the project. This group diversity has proven to be especially beneficial during the design phase because all ideas can be quickly and properly evaluated.

The ideal position would involve the purchase of off-the-shelf systems with all the necessary options and support. However, the author's group found a shortage of commercially available components necessary for the systems that we want. One of the luxuries that results from a highly skilled set of personnel is the ability to customize a system in virtually every facet. While this greatly increases the development time, the delivered system is seemingly a 'perfect fit' to the customer's automation needs.

### The move to 'smart systems'

Many of the earlier systems were showing signs of age and were in need of replacement. While simple at first, each subsequent system had become more advanced and complex than the previous. Each advancement was patched into the original, eventually culminating in a 'hodge-podge' algorithm. The timing seemed right to develop highly technical, dependable, and full-featured smart systems due to the existence of: robot friendly laboratories; experienced and highly skilled personnel; equipment and expertise to customize virtually any procedure; a comfort level (with respect to automation) among the development, customer, and management groups; and customers with experience in and enthusiasm for laboratory automation.

These new smart systems would include a number of design modifications and enhancements from previous systems including:

(1) *Modularity:* robots and their peripherals were placed on connecting tables suitable for quick-connect (tables, plumbing, electrical, data communications, etc.). This allowed the system to be disconnected, transported to a new location, then reconnected, all within a short time.
(2) *Error trapping:* the smart systems included over 70 error trappings. The robot attempts to recover from an error condition; should it fail after a number of attempts, the analysts are then notified by electronic page.
(3) *On-line help:* during an error condition, the computer monitor displays to the analyst the specific error with a maximum of three tips for correcting that error.
(4) *Data logging:* a multitude of data are recorded on floppy disk, including assay-specific data, any errors (with time stamp and location of the error), and error reset information (with time stamp and name of analyst correcting the error).
(5) *Sensors:* while previous systems had sensors, the smart systems include many more such as load cells and magnetic reed (photoelectric and proximity). These sensors ensure that moving devices and/or objects operate in the prescribed fashion.
(6) *Utilities:* again, previous systems included some utilities, but the smart systems contain a full set of utilities in order to 'test everything on the table', including every moving device and operation.

There were, it seemed, valid reasons for these enhancements, including the demand for more data, electronically

0142-0453/94 $10.00 © 1994 Taylor & Francis Ltd.

distributed results, self-documentation (for example, error occurrences, and system suitability checks), and greater control measures (measured liquid transfers, tactile feedback for object sensing, real-time monitoring of analytical data, etc.). In addition, with these added features and their associated data, the validation process should be greatly facilitated. Ozawa's [4] position was in line with ours: with greater intelligence, the system could better interact with the operations and recover (or at least attempt to recover) from error 'on-the-fly'.

As Brunner [5] has pointed out, it is tempting to develop systems for purpose of prestige or for the opportunity to work with state-of-the-art technology. However, our group was, seemingly, developing these smart systems for the right reasons because there was a business case. With the combination of both experience and objectivity, this ideology was destined to be a success and to advance itself in the future.

### The end result

The smart systems have performed as they were designed. However, the perception within the author's section is that they have failed to live up to their expectations. This perception is largely based on a time when poor performance existed due to the absence of system maintenance by the customer. (Failure to adhere to a daily maintenance routine is a prescription for trouble on any automated system, but principally with these smart systems. As an example, during one evaluation, approximately 30% of the errors could be associated with the lack of daily maintenance, for example, dirty fingers, o-rings, and syringes.) The poor performance was not a defect of the smart systems—the inability to give attention to daily maintenance has clearly caused numerous errors and enslaved the analysts to the robot. To achieve operational freedom, sensors were literally by-passed, or the system left in error (assuming the error condition did not halt robot operation). However, through this difficult period of adjustment, sample throughput continued and acceptable sample results were obtained.

Another reason for this perception of failure is the developer's worst nightmare: lack of customer buy-in. The customer was heavily involved in the design of these smart systems. With their experience came a comfort-level—an aggressiveness—in the new features these systems would employ. The sky was the limit and there were no fiscal barriers to these ideas. Perhaps the customer's comfort-level blinded the author's group to Hamilton's [6] advice: 'it cannot be realistically expected for a laboratory staff to cope overnight with a quantum leap of technology. . . . Such situations result in a very heavy support draining on the developing group, and usually generates discomfort with the user who feels loss of control of their process'. The customer experienced a much steeper learning curve than anticipated. To aid the customer in this learning endeavour, the author's group not only conducted training in much the same manner as on previous systems, but delivered an exhaustive set of manuals describing the system in immense detail. After delivering the product, every effort was made to minimize the learning curve and assist the customer in feeling comfortable with the smart systems. When it seemed the right time to let the customer use the system unaided, the system literally sat unused. While this was puzzling at first, perhaps the customer simply had a lack of comprehension due to the system's complexity. In retrospect, the author's group failed to match technology to the capabilities of the user.

After a time of frustration, a commitment was made by both groups to overcome these hurdles. Today, these systems are running numerous samples on a daily basis with good results. The enormous hurdles in getting these systems to the acceptance stage have been overcome and the customer is again speaking to us on friendly terms.

One unintended outcome is that the customer may be on the brink of information overload. These analysts must cope with faster turnaround, greater sample throughput, control and system suitability data, and system performance reports. Observation has shown these data to be largely unused. Reports are placed in piles and soon become buried under miscellaneous litter. It is the author's opinion that these data could increase daily performance by giving analysts the ability to be proactive, instead of reactive, to system events. However, the observed response should have been anticipated. On many occasions, additional tools are not fully utilized without a transformation in thinking. The conclusion is this: the failure to appropriately respond to these data has diminished their usefulness and has made the development of such items superfluous.

### A change in climate

The ideology of fabricating smarter, fully featured systems has died within the author's component. The new objective is to build a simpler system, yet one that still meets truly functional requirements. The drivers to this new ideology are many. In addition to the painful experiences previously discussed, the discussion below summarizes additional motives for building simpler systems:

(1) *Money:* simply stated, the cost of building smarter, fully featured systems is prohibitive. During these times of corporate pruning, building these systems is a veritable luxury unless there exists an *extremely strong* business case.
(2) *Time:* development time on these complex systems simply takes too many man-hours. The operative in today's pharmaceutical world is 'speed-to-market' and this ideology has permeated the author's company in virtually every facet.
(3) *Philosophy*: related to the dialogue above, the philosophy of the author's company is to focus on its 'core business'. The company can no longer afford to be in the *custom* robotics business. Unless there exists a solid business case, the development of custom components will be left to the third-party vendors. This philosophy to construct systems using commercially available equipment will be an enormous challenge. It will be necessary to give up features to accommodate the need to quickly develop systems. This challenge has forced the author's group to be innovative in designing systems. As Kramer [7]

pointed out, the future should bring more 'off-the-shelf' components that will obviously aid this new strategy. Although Hamilton [8] warned of the dangers in this strategy by stating that customization is the key to getting ahead, for the foreseeable future, developers within the author's company will continually examine their development efforts to ensure they are in line with corporate goals.

(4) *Robustness:* an added bonus to this new direction is that the simpler systems are more dependable and require less maintenance simply because they contain less-complicated features.

This change in ideology was implemented within the past year and has proven to be an immediate success. Within six months of project conception, a thoroughly tested and validated system was delivered. (Systems immediately prior to this took more than one year to develop and were not nearly as extensively tested.) How could system development time be reduced by more than 50%? One reason is that only the truly necessary items were automated. On-line help, many electronic and mechanical sensors, and system utilities were abolished. This enabled the author's group to reduce development time, development costs, and both the amount and complexity of system maintenance. There was, however, no compromise on system reliability. The system was built with the idea of thoroughly testing each component as it was completed. The result of this 'early and often' testing scheme was that over 800 samples were run through the system before system validation.

The days of fabricating a *totally automated* laboratory automation system are over, at least within the author's section. The group will automate only what is truly practical; instead of automating an entire process, only the 'bottleneck' of that process will be automated. This approach will require some manual operations. This would achieve another objective: give more responsibility to the user. As an example, some of the systems sensed the levels of waste vessels. While a convenience, these features were expensive and greatly added to the complexity of system development. The temptation is to ask more and more of systems and less and less of people.

The new systems are less sophisticated, and, at least on the surface, somewhat less user friendly. But, with less sophistication and complexity, the systems have proven to be more reliable and dependable. And ultimately, that is the best user friendly one can achieve!

There will be customers who are unable or unwilling to buy into this new strategy. There are numerous vendors that can fulfill that need. In fact, most competitors of the author's company have been using this strategy for years—and with favorable results. In the past, many of the author's customers have calculated the cost of their systems solely on the cost of acquiring or fabricating equipment. (The author's group does not charge other departments for its labour.) From a corporate standpoint, the total system cost is prohibitive when the cost of development, in both time and money, is included. The author's company can achieve true savings of resources by outsourcing these systems to vendors. In-house developers can then be used effectively elsewhere developing other systems that bring value to the company.

### The future

The author's group worked hard and delivered a product. The delivered product was a good one. But was it the *right* product? The new direction that the group is taking will ensure that the *right* product is developed—and in a more timely fashion. This strategy will enable the group to be more dynamic and respond quickly to changes and needs. Simpler systems are not only less expensive, but they have quicker development times. They greatly simplify the support and validation issues while still meeting their intended purpose.

With this new thinking has come new opportunities and challenges. The approach of automating only pieces and not processes has enlarged the contemplation of automation opportunities.

### References

1. Hamilton, S. D., *American Laboratory*, **19** (1987), 120.
2. Hamilton, S. D., *Journal of Automatic Chemistry*, **13** (1991), 25.
3. Brunner, L. A., *Journal of Automatic Chemistry*, **2** (1992), 44.
4. Ozawa, K., *Journal of Automatic Chemistry*, **14** (1992), 9.
5. Brunner, L. A., *Journal of Automatic Chemistry*, **14** (1992), 43.
6. Hamilton, S. D., *Journal of Automatic Chemistry*, **13** (1991), 24.
7. Kramer, G. W., *Clinical Chemistry*, **36** (1990), 1556–1560.
8. Hamilton, S. D., *Journal of Automatic Chemistry*, **13** (1991), 23.

